# The Late Miss Atkinson

**Published:** 1918-11-30

**Authors:** Margaret E. Shouler

**Affiliations:** General Hospital, Northampton.


### THE EDITOR'S LETTER-BOX—(*continued*).

### The Late Miss Atkinson.

*To the Editor of* THE HOSPITAL.

SIR,—On behalf of the mother and family of the late Miss Atkinson, matron of the General Hospital, Northampton, I desire personally through these columns to thank the governors of the hospital, the secretary, superintendent, and all the staff for their great kindness and genuine sympathy shown to my dear sister during her trying illness. She highly valued everything done for her, and was so happy and hopeful in her work at the hospital that it was a great grief that her stay was cut so short. Our heartfelt thanks go out to all concerned; the kindness of the Northampton people has indeed been beyond all comprehension, and it makes us realise the great appreciation of the dear departed's efforts and the many kind friends that she made.—Yours faithfully,

MARGARET E. SHOULER.

General Hospital, Northampton.
November 24, 1918.

